# The Treatment of Cutaneous Cancers*Abstract of paper read before the Tri-State Medical Society of Alabama, Georgia and Tennessee.

**Published:** 1896-02

**Authors:** A. R. Robinson

**Affiliations:** New York


ATLANTA

Medical and Surgical Journal.

VOL. XII. FEBRUARY, 1896. No. 12.

LUTHER B. GRANDY, M.D., MANAGING EDITOR.

M. B. HUTCHINS, M.D., BUSINESS MANAGER.

COLLABORATORS:
H. V. M. MILLER, M.D., LL.D., A. W. CALHOUN, M.D., LL.D., VIRGIL O. HARDON, M.D., FLOYD W. McRAE, M.D., AND DUNBAR ROY, M.D.

### *ORIGINAL COMMUNICATIONS.*

### THE TREATMENT OF CUTANEOUS CANCERS.*

By A. R. ROBINSON, M.D.,

NEW YORK.

Last year the author read a paper on the importance of early treatment of this disease, showing that the earlier a cancer was treated the more favorable is the prognosis and the less the deformity. The present paper discusses particularly the treatment, by appropriate caustics, the author claiming that in the great majority of cases much better results are obtained than by operations with the knife. Cancer can be regarded as primarily a local disease, and therefore a complete removal of the primary lesion before the disease has invaded other parts is equivalent to removal of the disease from the body. In all cases of epithelioma there is: 1st, an abnormal proliferation of a typical epithelium; 2d, this proliferation is associated with the production of a poison which injures surrounding tis-

*Abstract of paper read before the Tri-State Medical Society of Alabama, Georgia and Tennessee.

sues; 3d, epithelial invasion of the connective tissue by way of the lymphatics; and 4th, a tendency to secondary infection of the lymph glands.

He then described the manner in which an epithelioma extends, showing how insidiously the epithelium extends through the lymph channels in an irregular manner, and giving an area of extension far beyond what seems, with the naked eye, to be the limit of the growth. He next described the differences in the different clinical forms of epithelioma as regards the limit of probable extension at time of observation. Stress was laid upon the necessity of a correct diagnosis, not only as regards the form of the disease, but also of its actual existence, as he had too frequently seen serious surgical operations performed for cancer when antisyphilitic treatment would have promptly cured the cases. As some forms of cancer spread more rapidly than others, and the epithelial cells of some more mobile than those of others, it is also very necessary to be able to recognize the form of growth present.

Starting with the view that the method of removal that gives the best results, that which removes with the greatest certainty *all* of the morbid epithelial tissue is the preferable one, even if the resulting deformity or pain connected with the operation be greater than by other methods, and that of two methods of operation promising equal results, that causing least pain and least deformity should be employed, he discussed the various methods of treatment now employed.

As regards the use of the knife, it is clear from a study of the mode of origin, the method of extension and cause of recurrence in cancer ,that if the surgeon makes his incision beyond the limit of epithelial invasion there can be no recurrence of the disease. In the vast majority of cases of cutaneous epithelioma, however, such an operation causes much deformity and should not be employed in such cases when we have much better means for removal of the pathological tissue. There are, however, some parts of the body where the knife offers the greatest hope of cure. Upon the flexor surface of the forearm, near the wrist, an epithelioma sometimes develops, and unless seen very early, is best treated, in his experience, by amputation of the arm above the elbow. Also, cancer of the penis, in which the lymph gland or spongy tissue is already invaded, demands amputation, whilst other cases can be cured by caustics, especially the primary papillomatous form in an early stage. In epithelioma of the scrotum it is easy to remove a large amount of tissue without causing deformity, and in these cases the knife should be used and not caustics. On many parts, as forehead, cheek, lips, etc., it is not difficult to remove a considerable amount of tissue without producing much deformity, but on other parts, as the nose, this cannot be done, and in none of these cases should the knife be used, as for all cancers of the face other methods give better results. The author then described the usual method of procedure followed by the surgeon, and attacked the correctness of treating the wound in such a manner as to keep the part aseptic, holding that such a course gives more opportunity for recurrence than if suppuration is allowed to occur, as any pathological epithelium remaining is undisturbed and allowed to multiply, whereas, a suppurative inflammation would destroy, within a limited distance of the incision, this epithelium without destroying the normal tissue. If there is no question but that the incision includes all of the disease, then healing by first intention is correct; but experience shows that recurrences are the rule after a cutting operation. If the tumor is situated upon the nose and the growth be completely removed by incision, the consequent deformity is a serious objection to the operation and should not be resorted to, as other methods give better results. If the glands in the neck are invaded no operation should be performed, as all of the pathological tissue cannot be removed, and the disease is likely to grow with increased rapidity after unsuccessful surgical interference. The use of the knife is therefore limited to a small percentage of cases of cutaneous cancers.

Scraping or curetting are not reliable, as one cannot remove enough at a single operation, and in the intervals between the operations there is danger of secondary infection. Curetting is only useful to make a raw surface for a caustic paste in cases of superficial epithelioma.

The toxins of some organisms, especially those of erysipelas, have been employed by many surgeons, but in the author's experience are worthless as curative agents, and should never be used except as an experiment in absolutely hopeless cases. Any agent that makes a profound impression upon the whole economy has a tendency to cause a temporary diminution in the size of a growth depending upon organisms.

*Caustics.*—No caustic should be employed that does not quickly and effectually destroy, directly or indirectly, the pathological tissue. All the mild caustics, as nitric acid, nitrate of silver, etc., do more harm than good and should not be employed. The author gave in detail the reasons why a mild caustic caused the disease to spread with greater rapidity than before. The agents he employs are caustic potash, chloride of zinc, and arsenious acid. Caustic potash quickly liquefies tissue, and a small epithelioma can be destroyed at one sitting. The action of the caustic extends much beyond the area directly necrosed, causing an acute inflammation with much serous transudation. A toxalbumen is also probably formed, which acts upon any organisms, consequently by the use of caustic potash you obtain a curative action over a large area whilst completely necrosing only a small one. This is the advantage over the knife, and this advantage is so great that it should always be preferred except in the rare cases already mentioned. The amount to be completely destroyed should equal the apparent extent of the tumor but not beyond that. For early epithelioma of the lip, especially when of the papillomatous form, it is a very reliable agent. The author has removed large tumors on this part without producing any scar, and what is of importance, without recurrence of the disease. If the disease is on the nose arsenious acid is a better remedy as it leaves more normal tissue uninjured and hence causes less deformity.

Chloride of zinc can be used either in stick form or in solution or in paste. It does not destroy tissue as rapidly as caustic potash, and causes more pain, which also lasts for a longer period. It can be used in the same cases as caustic potash. Used as a paste it is a valuable adjunct for many cases. If 20 per cent. of cocaine be added to a Bougard's paste the pain is very slight and any desired amount of tissue can be removed. It causes a dry necrosis, so that after being applied for, say twenty hours, the dead tissue can be removed with a scalpel and a new application made and so on, until the entire growth is removed. It is best adapted to easily bleeding tumors, and in certain nodular growths of the scalp preparatory to the use of arsenious acid. It should not be used when it is desirable to save all the normal tissue possible, as it is but slightly elective in its action. It should not be employed in the superficial discoid form.

In arsenious acid in the form of a paste—Marsden's paste—we have an agent that is elective in its action and for the great majority of cases of cutaneous cancer should be employed in preference to all other means. It is useful in all forms of cutaneous cancers, and the results from its proper use are generally very satisfactory, both as regards cure and slight deformity. The paste consists of two parts by weight of arsenious acid and one part gum acacia, rubbed well together, and enough water then added to make a paste the consistence of butter. It should always, when possible, cover a surface half an inch beyond the apparent margin of the tumor, and left on from ten to eighteen or twenty hours, the duration depending upon the vulnerability of the tissues, and the extent of the disease, especially in depth. If the action has been sufficient, all of the area apparently occupied by the growth will have been necrosed and the surrounding tissue for a considerable distance in a condition of marked inflammatory edema. If such action has not taken place the paste must be reapplied the following day and again on the third day if necessary. The part is afterward treated by a simple ointment. This paste, or one composed of equal parts of arsenious acid and acacia is to be preferred in all cases where it is desirable to save normal tissue and have the least deformity after cure. It is the only application to be employed in cancer of the nose. If the tumor is situated just below the eye the lachrymal secretion may soften the paste too much or wash it away; in that case it may be necessary to apply a new paste several times during the ten to twenty hours. If situated near or on the lid a solution of cocaine can be dropped into the eye to lessen the pain.

For the successful use of the caustics several things are absolutely necessary. Their successful management is much more difficult than removal by the knife, but the results obtained are very much more favorable. The physician must be able to recognize the form of tumor, its probable area already invaded, and this differs much in the different forms of tumor, and the vulnerability of the tissues in each case. He must also recognize the manner in which the growth extends, and the factors favoring the extension. He must also know when the part has been sufficiently destroyed by the caustic, so that his treatment will be both effective and conservative. All these things demand pathological knowledge and an acquaintance with the histology and anatomy of the part affected. Finally, experience is necessary to obtain the best results. A proper appreciation of all these things, and the use of caustics instead of the knife, for suitable cases will, if employed, be the means of saving many lives and much suffering.

